# Human sperm ion channel (dys)function: implications for fertilization

**DOI:** 10.1093/humupd/dmz032

**Published:** 2019-10-30

**Authors:** Sean G Brown, Stephen J Publicover, Christopher L R Barratt, Sarah J Martins da Silva

**Affiliations:** 1 School of Applied Sciences, Abertay University, Dundee DD11HG, UK; 2 Systems Medicine, Ninewells Hospital and Medical School, University of Dundee, Dundee DD19SY, UK; 3 School of Biosciences, University of Birmingham B15 2TT, UK

**Keywords:** sperm, ion channels, CatSper, male infertility, calcium, potassium channel

## Abstract

**BACKGROUND:**

Intensive research on sperm ion channels has identified members of several ion channel families in both mouse and human sperm. Gene knock-out studies have unequivocally demonstrated the importance of the calcium and potassium conductances in sperm for fertility. In both species, the calcium current is carried by the highly complex cation channel of sperm (CatSper). In mouse sperm, the potassium current has been conclusively shown to be carried by a channel consisting of the pore forming subunit SLO3 and auxiliary subunit leucine-rich repeat-containing 52 (LRRC52). However, in human sperm it is controversial whether the pore forming subunit of the channel is composed of SLO3 and/or SLO1. Deciphering the role of the proton-specific Hv1 channel is more challenging as it is only expressed in human sperm. However, definitive evidence for a role in, and importance for, human fertility can only be determined through studies using clinical samples.

**OBJECTIVE AND RATIONALE:**

This review aims to provide insight into the role of sperm ion channels in human fertilization as evidenced from recent studies of sperm from infertile men. We also summarize the key discoveries from mouse ion channel knock-out models and contrast the properties of mouse and human CatSper and potassium currents. We detail the evidence for, and consequences of, defective ion channels in human sperm and discuss hypotheses to explain how defects arise and why affected sperm have impaired fertilization potential.

**SEARCH METHODS:**

Relevant studies were identified using PubMed and were limited to ion channels that have been characterized in mouse and human sperm. Additional notable examples from other species are included as appropriate.

**OUTCOMES:**

There are now well-documented fundamental differences between the properties of CatSper and potassium channel currents in mouse and human sperm. However, in both species, sperm lacking either channel cannot fertilize *in vivo* and CatSper-null sperm also fail to fertilize at IVF. Sperm-lacking potassium currents are capable of fertilizing at IVF, albeit at a much lower rate. However, additional complex and heterogeneous ion channel dysfunction has been reported in sperm from infertile men, the causes of which are unknown. Similarly, the nature of the functional impairment of affected patient sperm remains elusive. There are no reports of studies of Hv1 in human sperm from infertile men.

**WIDER IMPLICATIONS:**

Recent studies using sperm from infertile men have given new insight and critical evidence supporting the supposition that calcium and potassium conductances are essential for human fertility. However, it should be highlighted that many fundamental questions remain regarding the nature of molecular and functional defects in sperm with dysfunctional ion channels. The development and application of advanced technologies remains a necessity to progress basic and clinical research in this area, with the aim of providing effective screening methodologies to identify and develop treatments for affected men in order to help prevent failed ART cycles. Conversely, development of drugs that block calcium and/or potassium conductances in sperm is a plausible strategy for producing sperm-specific contraceptives.

## Introduction

Infertility is a global health challenge, estimated to affect ~10–13% of people of reproductive age (Datta *et al.,* 2016). Sperm dysfunction, particularly impaired motility, is a significant contributing factor. However, no drugs are available to rescue sperm function *in vivo* or *in vitro*. A fundamental reason for this is a lack of understanding of the cellular and molecular control of the physiological functions of spermatozoa (Barratt *et al.,* 2017). It is well over a decade since the first identification of the cation channel of sperm (CatSper) in the plasma membrane of mouse sperm. Numerous studies have subsequently characterized ion channels in mammalian sperm, including sperm from fertile men and patients undergoing ART. It has become clear that ion channel activities are crucial for sperm function and that dysfunction of these channels has significant consequences for fertilization. This review re-evaluates proposed models for the physiological role(s) of sperm ion channels and discusses important issues that arise from recent studies of sperm from infertile patients.

## Methods

A comprehensive literature search was undertaken using the PubMed database to find articles up to May 2019 that reported on studies investigating human and mouse sperm ion channels. Notable examples from other species were added as required to complete the narrative. The search terms included, either alone or in combination with sperm, were CatSper, calcium signalling, ion channels, potassium channel, infertility and membrane potential.

## CatSper

### The discovery and importance of CatSper for fertilization

Early investigations into the regulation of sperm functions established calcium signalling as a regulator of sperm acrosome reaction and motility (Placzek *et al.,* 1987; Thomas and Meizel, 1988; Mortimer *et al.,* 1988; Stock and Fraser, 1989; Serres *et al.,* 1991; Blackmore, 1993). In human sperm, progesterone (P4), prostaglandin (PG) E1 and E2 were noted as potent agonists of these functions through their capacity to rapidly elevate intracellular calcium ion concentration ([Ca^2+^]_i_, Aitken *et al.,* 1986; Colon *et al.,* 1986; Shimizu *et al.,* 1988; Blackmore *et al.,* 1991; Yang *et al.,* 1994; Schaefer *et al.,* 1988, 2000). The extracellular site of action of P4 (Blackmore and Lattanzio 1991; Blackmore *et al.,* 1991; Meizel and Turner, 1991), the inhibitory effect of extracellular calcium chelation and efficacy of some calcium channel blockers on sperm function and progesterone-induced calcium influx (Placzek *et al.,* 1987; Blackmore *et al.,* 1990; Foresta *et al.,* 1993; Kanwar *et al.,* 1993; Anand *et al.,* 1994; O’Toole *et al.,* 1996; Goodwin *et al.,* 1997) supported the hypothesis of involvement of an ion channel in these processes (Shimizu *et al.,* 1988; Blackmore *et al.,* 1990).

**Figure 1:**
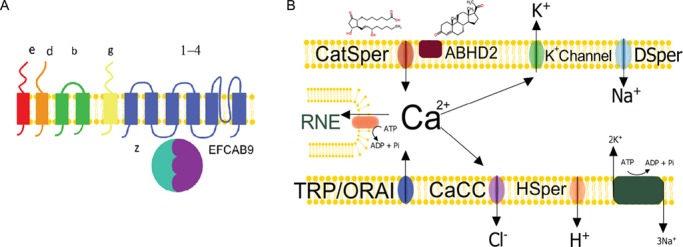
**Human sperm ion channels.** (**A**) Cartoon showing the topology of the pore forming (1–4) and auxiliary subunits that contribute to the mature CatSper complex. The recently described EFCAB9 is an evolutionary conserved CatSper auxiliary subunit that possesses three EF-hand calcium-binding domains and interacts with CATSPER ZETA to regulate channel conductance in mouse sperm (Hwang *et al.,* 2019). (**B**) Hypothetical model for human sperm ion channel function in the principal piece of the flagellum. Extracellular calcium entry has been definitely shown to occur through CatSper, which is therefore implicated in the regulation of sperm function. Elevated [Ca^2+^]_i_ may also be achieved through release from intracellular Ca^2+^ stores located in the RNE. CatSper and additional Ca^2+^-conducting membrane channels such as TRP and/or calcium release-activated calcium channel protein (ORAI) may contribute to refilling the intracellular store. The cyclical release and refilling of these stores causes intracellular Ca^2+^-oscillations that are likely to influence motility and the acrosome reaction. CatSper activity is negatively regulated by endogenous endocannabinoids such as 2AG. Progesterone (P4) is proposed to activate CatSper indirectly through activation of the plasma membrane bound lipid hydrolase ABHD2, which metabolizes 2AG. In contrast, prostaglandin E1 (PGE1) may activate the channel directly. CatSper is also regulated by membrane potential (Vm) and pH_i_. Entry of a depolarizing current ‘DSper’ through TRPV4 may facilitate CatSper activation. Depolarization-activated Hv1 (and/or HSper) channels contribute to elevating intracellular pH by permitting proton efflux that facilitates CatSper function. Hv1 is inhibited by extracellular Zn^2+^ and potentiated by anandamide. Hyperpolarization/repolarization is regulated by the Ca^2+^-sensitive sperm potassium channel and chloride channel (CaCC) as well as sodium/potassium ATPase. Therefore, the K^+^ channel (SLO3/LRRC52) and CaCC may have a reciprocal relationship with CatSper. Elevated [Ca^2+^]_i_ upon stimulation of CatSper may activate the sperm K^+^ channel. The resulting hyperpolarization may act to reduce CatSper opening. [Ca^2+^]_i_ can be lowered by plasma membrane Ca-ATPase activity and SERCA. Conversely, activation of CaCC may facilitate CatSper opening through depolarization of Vm. Furthermore, high concentrations of P4 block I_KSper_ which may facilitate maximal activation of CatSper close to the cumulus-oocyte-complex. However, the nature of interplay between these channels, resultant sperm behaviours and *in vivo* activity currently remains elusive. We have demonstrated that loss of I_KSper_ does not impact on CatSper expression/P4-efficacy but does significantly impact on fertilizing capacity at IVF. In normal sperm, opening of CaCC will cause depolarization. We hypothesize that dysfunctional sperm with unusually depolarized Vm opening of CaCC will cause hyperpolarization, which may help to prevent total fertilization failure in IVF. It is unclear if sperm from patients with unusually high resting [Ca^2+^]_i_ is due to elevated basal CatSper conductance or suboptimal extrusion.

Despite molecular and immunohistochemical analysis of spermatocytes and sperm from various species providing evidence in support of the expression of a number of candidate channels (Weyand *et al.,* 1994; Santi *et al.,* 1996; Wiesner *et al.,* 1998; Serrano *et al.,* 1999; Wennemuth *et al.,* 2000), neither the nature of the sperm membrane Ca^2+^ channel(s) nor its regulation by P4 in human sperm could be further characterized due to technical challenges of applying patch-clamp electrophysiology to whole sperm. Initial attempts at characterization of channels in boar, sea urchin and mouse sperm relied on fusion of the cell membrane to planar bilayers (Guerrero *et al.,* 1987; Cox *et al.,* 1991; Babcock *et al.,* 1992; Beltrán *et al.,* 1994; Labarca *et al.,* 1996; Tiwari-Woodruff *et al.,* 1995). Single-channel recordings from mouse and human sperm head were achieved but did not yield clear information on a sperm Ca^2+^ channel (Espinosa *et al.,* 1998; Gu *et al.,* 2004; Jiminez-Gonzalez *et al.,* 2007). A breakthrough came upon the identification of CatSper, a novel testis-specific channel, and the study of genetically modified CatSper-null mice. Animals that lack CATSPER1 (one of the four subunits that make up the channel pore) are infertile and have sperm that exhibit motility defects and fail to fertilize at IVF. CatSper, which localizes to the principle piece of the flagellum, was deemed critical for sperm motility and male fertility (Ren *et al.,* 2001). Despite considerable effort, heterologous expression of functional CatSper *in vitro*, allowing electrophysiological characterization, proved (and currently remains) impossible. A significant reason for this is that CatSper is a highly complex multi-subunit ion channel made up of at least 10 subunits (Quill *et al.,* 2001, 2003; Ren *et al.,* 2001; Carlson *et al.,* 2003; Lobley *et al.,* 2003; Liu *et al.,* 2007; Qi *et al.,* 2007; Wang *et al.,* 2009; Chung *et al.,* 2011, 2017; Hwang *et al.,* 2019). CATSPER 1-4 alpha-subunits form the channel pore and are essential for formation of a functional channel complex. Knock-out of any of these subunits prevents functional channel expression and completely compromises fertilization *in vivo* and *in vitro*. CATSPER BETA, DELTA, GAMMA, EPSILON, ZETA and EFCAB9 are accessory subunits (Fig. 1A) whose expression in sperm is dependent upon that of CATSPER 1 (Chung *et al.,* 2011, 2017; Hwang *et al.,* 2019). Accessory subunit CATSPER DELTA-null male mice are infertile and their sperm fail to express the mature CatSper (Chung *et al.,* 2011). Genetic disruption of CATSPER ZETA or EFCAB9 expression causes subfertility and reduced CatSper expression within disorganized CatSper signalling domains (Chung *et al.,* 2017; Hwang *et al.,* 2019). Importantly, genomic errors at CatSper subunit loci are reported in infertile men (Avidan *et al.,* 2003; Avenarius *et al.,* 2009; Bhilawadikar *et al.,* 2013; Jaiswal *et al.,* 2014; Brown *et al.,* 2018; Luo *et al.,* 2019). Therefore, there is significant genetic evidence to argue that CatSper is essential for male fertility in mouse and humans. CatSper subunits are evolutionary conserved in a large number of genomes of Eumetazoa (Cai and Clapham, 2008; Cai *et al.,* 2014). It has even been demonstrated that CatSper controls chemotaxis of sea urchin sperm (Seifert *et al.,* 2015) and may play a role in the fertilization competence of sperm from some fish species (Yanagimachi *et al.,* 2017). Despite the evolutionary conservation the subunit homology difference between mouse and human (CATSPER 1 50% identical, CATSPER 2 62%, CATSPER 3 66%, CATSPER 4 69%, CATSPER BETA 57%, CATSPER DELTA 51%, CATSPER GAMMA 57%, CATSPER EPSILON 52%, CATSPER ZETA 46%, EFCAB9 60%. Source: https://www.uniprot.org/) suggests the potential for significantly divergent function and/or regulation. Indeed, the weight of evidence now suggests that mouse CatSper is not a reliable surrogate for studying channel function in human sperm (Kaupp and Strunker, 2017 and see below). It is therefore necessary to study sperm from fertile and infertile men to determine the role and importance of CatSper for human fertilization.

### The characteristics and function of human CatSper

It was shown almost 30 years ago that application of P4 to human sperm evokes an ‘immediate’ Ca^2+^ influx and consequent increase in [Ca^2+^]_i_ (Blackmore *et al.,* 1990), and this ‘non-genomic’ action of the steroid can be considered a dogma of human sperm physiology (Blackmore *et al.,* 1991; Baldi *et al.,* 1991; Foresta *et al.,* 1993; Plant *et al.,* 1995; Meizel *et al.,* 1997; Kirkman-Brown *et al.,* 2000, 2002, 2004; Harper *et al.,* 2003, 2004, 2006; Bedu-Addo *et al.,* 2007; Strunker *et al.,* 2011; Brenker *et al.,* 2012; Servin-Vinces *et al.,* 2012; Alasmari *et al.,* 2013b; Sánchez-Cárdenas *et al.,* 2014; Rennhack *et al.,* 2018). P4 is highly potent (EC_50_ = 10±10nM, Strunker *et al.,* 2011) and elevates [Ca^2+^]i in the great majority of cells (typically >90% of the observed sample, Meizel *et al.,* 1997; Kirkman-Brown *et al.,* 2000; Harper *et al.,* 2003; Kelly *et al.,* 2018). However, the molecular identity of this P4-activated channel became apparent only upon the successful application of whole-cell patch clamp electrophysiology to sperm. Whole-cell recordings were first obtained from mouse sperm. Inward cation currents present in the tails of wild-type sperm were absent in sperm from CatSper-null mice, showing that the recorded currents were carried by CatSper (Kirichok *et al.,* 2006). Application of the technique to human sperm revealed similar currents (I_CatSper_), also localized to the flagellum. However, although the current characteristics resembled those of mouse I_CatSper_, human I_CatSper_ is strongly potentiated by P4 and PGE1. Comparison of the characteristics of P4-induced Ca^2+^ influx and potentiation of I_CatSper_ confirmed that the action of P4 on human sperm is mediated by activation of CatSper (Lishko *et al.,* 2011; Strunker *et al.,* 2011).

Mouse sperm are much less sensitive to P4 than human, an increase in [Ca^2+^]_i_ being observed only with 10–100 μM P4 (10^3^× higher than EC_50_ for human sperm) and even then only ~40% of sperm respond (Kobori *et al.,* 2000; Romarowski *et al.,* 2016). Notably, the ability of high concentrations of P4 to evoke Ca^2+^ signals in mouse sperm is not affected by *Catsper 1*-knockout (Ren *et al.,* 2001) confirming that this effect is exerted through a completely different mechanism to that observed in human cells. However, human and mouse CatSper do have phenotypic similarities. For example, in both species the channel is activated at alkaline pH, is weakly voltage sensitive and the pore region has a high affinity for divalent ions (as in mouse sperm, human I_CatSper_ is almost exclusively recorded being carried by monovalent ions under divalent-free conditions; Lishko *et al.,* 2011; Strunker *et al.,* 2011). However, under these conditions there is a stark contrast between mouse and human I_CatSper_: mouse I_CatSper_ is large and quasilinear (Kirichok *et al.,* 2006), whereas inward human I_CatSper_ is smaller and outwardly rectifying. Only when challenged with P4 does human I_CatSper_ resemble that of mouse (Lishko *et al.,* 2011). Analysis of human CatSper conductance under divalent conditions illustrates that P4 and capacitation act independently and co-operatively to change the voltage sensitivity of the channel such that it is open at more physiologically relevant negative potentials (<0mV, Lishko *et al.,* 2011). It has been proposed that the low basal I_CatSper_ is due to suppression of channel activity by endogenous plasma membrane endocannabinoids, which are metabolized by a P4-sensitive membrane lipid hydrolase (ABHD2) present in the principal piece (Miller *et al.,* 2016; Fig. 1B). Application of exogenous endocannabinoids, namely 2-arachidonoylglycerol (2AG) and 1-arachidonoylglycerol, abolish human I_CatSper_ while they only suppress mouse I_CatSper_. Interestingly, enzyme inhibition does not affect PGE1 sensitivity suggesting that it may activate the channel directly (Miller *et al.,* 2016). Collectively, these studies demonstrate that mouse and human CatSper are regulated in fundamentally different ways and that both the female tract and membrane potential can influence human sperm function by elevating [Ca^2+^]_i_ through activation of CatSper.

Given the genetic and biophysical evidence of the prominence of CatSper in sperm, fundamental questions arise, for example what is the physiological function of CatSper in human sperm, and what is the consequence for male fertility for human sperm that lack CatSper? Loss of, or a diminished, response to P4 has been documented in sperm from patients attending a fertility clinic (Falsetti *et al.,* 1993; Oehninger *et al.,* 1994; Krausz *et al.,* 1995, 1996; Espino *et al.,* 2009; Alasmari *et al.,* 2013a; Williams *et al.,* 2015; Brenker *et al.,* 2018; Luo *et al.,* 2019). Furthermore, the P4-induced [Ca^2+^]_i_ response is correlated with IVF outcome (Krausz *et al.,* 1995, 1996), implying that the P4 response (or the mechanism that mediates it) could be critical for fertilization. In mouse sperm, CatSper-mediated Ca^2+^ entry appears to be crucial for regulation of motility. Sperm from CatSper-null mice show impaired motility and are unable to hyperactivate, which compromises both their ability to ascend the female tract and to penetrate the zona (Carlson *et al.,* 2003; Ho *et al.,* 2009). In human sperm, P4 stimulates cell penetration into viscous medium (mimicking penetration into cervical mucus and/or cumulus matrix), suggesting a similarly important role for human CatSper in regulating sperm motility (Alasmari *et al.,* 2013a, 2013b; Rennhack *et al.,* 2018). However, the importance of P4-induced CatSper activation in hyperactivated motility of human sperm is less clear. Assessment of flagellar activity in loosely tethered human sperm shows both increased flagellar bending and beat asymmetry upon exposure to P4, consistent with induction of hyperactivation (Harper *et al.,* 2004; Servin-Vences *et al.,* 2012; Schiffer *et al.,* 2014; Saggiorato *et al.,* 2017). However, although many authors have observed that P4 increased the percentage of hyperactivated sperm within a population (Mbizvo *et al.,* 1990; Uhler *et al.,* 1992; Yang *et al.,* 1994; Calogero *et al.,* 1996; Teves *et al.,* 2006; Kilic *et al.,* 2009; Armon and Eisenbach, 2011; Alasmari *et al.,* 2013b; Rennhack *et al.,* 2018), the reported effects are inconsistent (see Kay *et al.,* 1994; Emiliozzi *et al.,* 1996) and often very small (<10% of cells recruited into the ‘hyperactivated population’ at saturating P4 concentration). It is likely that the timing of motility assessment is crucial. When P4 is applied to human sperm as a bolus (stepped increase in concentration), the [Ca^2+^]_i_ signal is composed of a transient, which lasts only 30–60 s at 37^°^C, followed by a smaller plateau or slowly increasing ramp (Strunker *et al.,* 2011). Observation of sperm activity immediately upon P4 application (during the transient) shows a marked increase in flagellar activity and asymmetry (Servin-Vences *et al.,* 2012; Smith *et al.,* 2013; Schiffer *et al.,* 2014; Saggiorato *et al.,* 2017). During [Ca^2+^]_i_ imaging experiments on immobilized human sperm, we have often observed a striking but brief increase in flagellar activity during the [Ca^2+^]_i_ transient (e.g. Bedu-Addo *et al.,* 2007). However, when assessment of motility is made using computer-assisted sperm analysis (CASA), the delay required to transfer cells to the chamber after addition of P4 is such that measurements are likely to be made when the transient has partially (or fully) subsided. It is likely that the relatively small effects of P4 measured in many CASA studies reflect this issue. Other manipulations, such as use of 4-aminopyridine and elevation of extracellular pH, are considerably more potent when hyperactivation is assessed by CASA, apparently because a significant increase in [Ca^2+^]_i_ is maintained for an extended period (Alasmari *et al.,* 2013a, b; Achikanu *et al.,* 2018).

Assessment of the role of CatSper in activities of human sperm is complicated by the presence of an intracellular calcium store in the region of the redundant nuclear envelope (RNE) that is proposed to contribute to generation of Ca^2+^ signals. Cyclical mobilization and refilling of this Ca^2+^ store contributes to intracellular Ca^2+^ oscillations that are seen following the P4-induced (CatSper-mediated) [Ca^2+^]_i_ influx (Fig. 1B), though these oscillations require the presence of extracellular calcium (Harper *et al.,* 2004). [Ca^2+^]_i_ oscillations and ‘spiking’ can also occur spontaneously (particularly in cells capacitated at low pH; Mata-Martinez et al, 2018). A model of CatSper-dependent calcium-induced calcium release has been proposed (Harper *et al.,* 2004; Alasmari *et al.,* 2013b; Sánchez-Cárdenas *et al.,* 2014; Mata-Martinez et al, 2018). P4-induced [Ca^2+^]_i_ oscillations are associated with increased flagellar excursion (Harper *et al.,* 2004; Bedu-Addo *et al.,* 2007) and both spontaneous and P4-induced oscillations appear to inhibit the acrosome reaction (Harper *et al.,* 2004; Sánchez-Cárdenas *et al.,* 2014; Mata-Martinez et al, 2018). However, the percentage of acrosome reacting cells (~10%, Harper *et al.,* 2008; Sánchez-Cárdenas *et al.,* 2014; Mata-Martinez et al, 2018) is much less than the proportion of cells that respond to P4 but do not display intracellular calcium oscillations (≥60%, Harper *et al.,* 2004; Kelly *et al.,* 2018; Mata-Martinez et al, 2018). A possible explanation is that P4 (and follicular fluid) has a priming role for zona pellucida-induced acrosome induction (Schuffner *et al.,* 2002).

Therefore, the evidence from physiological and pharmacological studies supports a substantive role of CatSper in human sperm function. However, pharmacological intervention to interrogate the role of CatSper in human sperm cell function is complicated by the curious ligand sensitivity of this channel. A wide variety of small organic molecules activate the channel by mechanisms which mimic that of P4 or PGE1 (Brenker *et al.,* 2012; Tavares *et al.,* 2013; Schiffer *et al.,* 2014; Rehfeld *et al.,* 2016; Martins da Silva *et al.,* 2017), and I_CatSper_ is blocked by a bewildering array of chemically unrelated compounds (Brenker *et al.,* 2014; Mansell *et al.,* 2014; He *et al.,* 2016; Mannowetz *et al.,* 2017). Indeed, antagonists that are used to interrogate CatSper function (NNC 55-0396, mibefradil, MDL12330A) also show an inhibitory effect on the K^+^ current (Lishko *et al.,* 2011; Strunker *et al.,* 2011; Brenker *et al.,* 2012, 2014; Rennhack *et al.,* 2018). Remarkably, NNC 55-0396 and mibefradil, two compounds that have been shown to block current through CatSper, can elevate intracellular pH, increase [Ca^2+^]i and induce the acrosome reaction in human and mouse sperm (Chavez *et al.,* 2018). Data from experiments in which these blockers are used to target CatSper must be interpreted cautiously. To definitively answer questions on CatSper function, studies must be undertaken using sperm from natural CatSper ‘knock-out’ men.

### Evidence for and consequences of impaired CatSper function in human sperm

**Figure 2:**
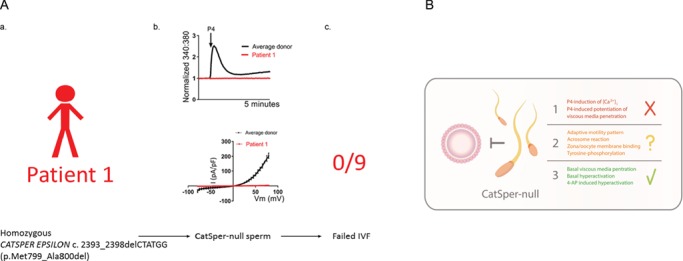
**Observations from studies of CatSper-null human sperm.** **A**. A diagrammatic summary of our findings from the study of patient 1 in Brown et al. (2018). (a) A microdeletion in exon 18 of the CatSper auxiliary subunit epsilon gene (*CATSPER EPSILON*) is associated with loss of CatSper function in sperm, which results in infertility. Patient 1 is homozygous for a 6-base pair in frame deletion in exon 18 of *CATSPER EPSILON,* which results in a loss of Methionine799 and Alanine800 in the putative extracellular domain of CATSPER EPSILON protein. This results in production of I_CatSper_-null sperm, which do not have any overt signs of sperm dysfunction (sperm concentration and motility were normal). (b) P4 fails to elicit an increase in [Ca^2+^]_i_ (upper panel) in cell populations and I_CatSper_ was confirmed to be absent by electrophysiology (lower panel). Data are those presented previously (Williams *et al.,* 2015). (c) The consequence of loss of CatSper function is failed fertilization (0 out of 9 eggs inseminated) at IVF. **B**. Questions and answers regarding human sperm function that lack functional CatSper. I_CatSper_-null human sperm cannot fertilize at IVF. From our study (using cells from the same sperm population that failed IVF), we demonstrate that P4 could not induce an increase in [Ca^2+^]_i_ or increase penetration into viscous media above basal levels (red). The number of cells exhibiting hyperactivation was within a normal range and could be boosted by treatment with 4-aminopyridine (4-AP). Molecular and cellular impairment that may arise due to CatSper dysfunction may include abnormal tyr/ser/thr-phosphorylation, deficiency of adaptive motility patterns, loss of zona/egg binding and failure of the acrosome reaction (amber), as suggested by Luo et al. (2019).

Unlike in the mouse, the functional significance of human CatSper cannot be tested by genetic manipulation. However, screening of men attending for infertility treatment may reveal the occurrence of ‘natural knockouts’ that affect sperm and/or male fertility. In fact, it has been known for some time that failed or significantly impaired [Ca^2+^]_i_ responses to P4 are prevalent in sperm used for IVF and ICSI (Falsetti *et al.,* 1993; Krausz *et al.,* 1995, 1996), though the significance of this with regard to CatSper function has only recently been recognized (Alasmari *et al.,* 2013b; Tamburrino *et al.,* 2014, 2015; Marchiani *et al.,* 2017; Luo *et al.,* 2019). Only the application of patch clamp electrophysiology, in a limited number of cases, has definitely answered whether P4-insensitive sperm lack I_CatSper_. The first description of CatSper null sperm was that from three brothers who had a complex medical history of congenital dyserythropoietic anemia type 1, deafness and infertility (Avidan *et al.,* 2003). In addition to the causative mutation for congenital dyserythropoietic anemia type 1, the men were also homozygous for an ∼70 kb deletion of the proximal copy of a 106 kb tandem repeat on chromosome 15q15 affecting four genes, including *CATSPER2*. Semen parameters were abnormal as the men have asthenoteratozoospermia. However, the absence of I_CatSper_ was subsequently confirmed using sperm from one of the affected men (Avidan *et al.,* 2003; Smith *et al.,* 2013). Similar clinical cases of men with infertility and differing degrees of hearing impairment from three Iranian families were reported (Zhang *et al.,* 2007). Although the chromosomal breakpoints differed between families, they shared a common homozygous autosomal recessive contiguous gene deletion affecting an ~100 kb region on chromosome 15q15 that included *CATSPER2*. Semen parameters were consistent with asthenoteratozoospermia (Zhang *et al.,* 2007). Avenarius et al. (2009) reported non-syndromic male infertility from two Iranian families who carried two separate insertion mutations (c.539-540insT and c.948-949insATGGC) in *CATSPER1* that are predicted to lead to frameshifts and premature stop codons (p.Lys180LysfsX8 and p.Asp317MetfsX18). Semen parameters were again consistent with asthenoteratozoospermia (Avenarius *et al.,* 2009). However, interpretation of the role of CatSper in male fertility from such studies is confounded by coexisting defects in spermatogenesis and other issues that potentially contribute to fertilization failure. An indirect method of identifying patients with CatSper-null sperm is to use the P4-induced [Ca^2+^]_i_ response to screen sperm from patients undergoing ART (either on the day of treatment or following failed/low fertilization) followed by patch clamp analysis. Using this approach, two cases were observed where sperm failed completely to respond to P4, and IVF was simultaneously affected by failed fertilization (Williams *et al.,* 2015). Sperm from Patient 1 showed no [Ca^2+^]_i_ response to P4 on two separate occasions and using electrophysiology I_CatSper_ was confirmed to be absent. Exome analysis did not reveal any loss of function mutations in CatSper subunit loci nor in any other genes known to be associated with failed fertilization in male mouse models (Williams *et al.,* 2015). However, following the discovery of *CATSPER EPSILON* and *ZETA*, exome sequences for patient 1 were re-interrogated, revealing a homozygous 6-base pair microdeletion in exon 18 of *CATSPER EPSILON* (Brown *et al.,* 2018; Fig. 2A). Sperm from patient 2 was studied on the day of treatment and P4 elicited no [Ca^2+^]_i_ and zero out of nine eggs were fertilized. Intriguingly, however, when sperm from this patient was studied 7 months later (on the day of ICSI treatment) P4 elicited a normal increase in [Ca^2+^]_i_ and fertilization was achieved. In stark contrast to mouse CatSper-null sperm, there were no gross abnormalities in sperm motility in samples that were refractory to P4 although in these cases P4 did not stimulate penetration into viscous media, thus demonstrating physiological impairment. Hyperactivation induced by pharmacological mobilization of Ca^2+^ from intracellular stores was normal in sperm cell samples from both patients (Williams *et al.,* 2015). Recently, Luo et al. (2019) described a case of a novel copy number variant in 15q15 (deafness–infertility locus), which resulted in a heterozygous ~55Kb deletion causing loss of entire *CATSPER2*. Intriguingly, semen parameters were normal but I_CatSper_ was absent and sperm function was impacted as reported previously for the *CATSPER EPSILON* deletion (Brown *et al.,* 2018). The reason for the profound effect of the heterozygous deletion remains to be established but may be related to mutations in additional gene regulatory elements (Luo *et al.,* 2019).

These studies suggest that CatSper is required for fertilization success in humans and that lesions in CatSper function/expression can arise because of genetic and/or channel biogenesis defects during spermatogenesis, which in some cases may be transient. Indeed, we also reported (and have subsequently observed one other case) an episodic loss of the P4-induced [Ca^2+^]_i_ signal in sperm from a donor. Moreover, CatSper may be critical for transit through the female tract to the site of fertilization (Suarez *et al.,* 2008) as has been definitively shown in mouse where CatSper is necessary for transition from the uterus into the oviduct (Chung *et al.,* 2014). However, the exact nature of functional deficiencies in CatSper-null human sperm remains to be conclusively established (Fig. 2B). In order to develop strategies to rescue function, it is fundamentally important to understand channel biogenesis, the role of intracellular Ca^2+^ stores and the contributions to sperm function of agonists, in addition to P4, that elevate [Ca^2+^]_i_ (Morales *et al.,* 2000; Brown *et al.,* 2018).

## The sperm potassium channel

### The discovery and importance for fertilization

Potassium channels play an integral role in setting and restoring cell membrane potential (Vm) and, in doing so, influencing the activity of voltage-sensitive ion channels. Using voltage-sensitive dyes, it has been calculated that the Vm of non-capacitated human spermatozoa is ~−40 mV (Linares-Hernández *et al.,* 1998) and that capacitation induces a negative shift in Vm (hyperpolarization) to ~−60/−70 mV (Patrat *et al.,* 2002). Early studies into the effects of elevating extracellular K^+^ concentration (to shift E_K_ to more positive values) on human sperm function give an insight into the consequences of membrane potential changes. Increasing extracellular K^+^ from standard levels (typically ≈5 mM) to concentrations reported in tubular fluid (25–30 mM, Borland *et al.,* 1980) induces an increase in [Ca^2+^]_i_ in non-capacitated and capacitated cells that is further potentiated by P4 (Linares-Hernández *et al.,* 1998; Kumar *et al.,* 2000) and increases the percentage of acrosome reacted sperm (Roblero *et al.,* 1988). Thus K^+^ channel activity may regulate sperm function indirectly through an increase of [Ca^2+^]_i_. Attempts to identify the K^+^ channel type(s) by pharmacological assessment have been hampered by the poor selectivity of the available reagents and, as a consequence, a number of candidates have been suggested (see for example, Yeung and Cooper, 2001; Barfield *et al.,* 2005). Similarly, several different K^+^ channel families have been immunolocalized to testis and/or mature sperm (Salvatore *et al.,* 1999; Jacob *et al.,* 2000; Munoz-Garay *et al.,* 2001; Yeung and Cooper, 2001; Acevedo *et al.,* 2006; Barfield *et al.,* 2005; Yi *et al.,* 2011). However, recent studies using patch clamp electrophysiology and genetically modified mouse models have made significant progress in determining the molecular nature of the sperm K^+^ conductance.

### The characteristics and function of the human sperm potassium channel

The biophysical characteristics of the sperm K^+^ conductance were first described using mouse sperm under whole-cell patch clamp conditions. Alkalinisation activates a pH- and voltage-sensitive outwardly rectifying K^+^ current (I_KSper_) in the principle piece to cause rapid hyperpolarization of the membrane potential (Navarro *et al.,* 2007; MartÍnez-López *et al.,* 2009). Electrophysiological studies of sperm from normal and genetically modified mice confirmed that the pore forming subunit Slo3 (KCNU1) is present in the principle piece and constitutes the K^+^ channel responsible for mouse I_KSper_ and is necessary for fertility. Mutant male mice are infertile and their sperm give very poor fertilization rates at IVF (Santi *et al.,* 2010; Zeng *et al.,* 2011). However, the voltage sensitivity of heterologously expressed SLO3 channels is different to that of I_Ksper_, suggesting that an additional regulatory component is present in sperm (Zhang *et al.,* 2006; Navarro *et al.,* 2007). Only by co-expression with the auxiliary subunit leucine-rich repeat-containing 52 (LRRC52) do the biophysical characteristics of expressed mouse Slo3 closely resemble those of native mouse I_KSper._ LRRC52 is necessary in order to permit the channel to activate at physiologically relevant voltages and pH values (Yang *et al.,* 2011; Leonetti *et al.,* 2012; Zeng *et al.,* 2015). The voltage sensitivity of I_KSper_ in sperm from LRRC52-null mice is severely reduced, which causes male sub-fertility and low IVF success rates (Zeng *et al.,* 2015). Thus, the normal fertilizing potential of mouse sperm is dependent upon expression of the heteromeric K^+^ channel SLO3/LRRC52, which permits pH regulation of Vm.

In sharp contrast, human I_KSper_ is regulated by [Ca^2+^]_i_, only relatively weakly activated by alkalinisation and blocked by high concentrations (≈30 μM) of P4 (Mannowetz *et al.,* 2013; Brenker *et al.,* 2014; Mansell *et al.,* 2014). Determining the nature of the K^+^ conductance in human sperm has not been without controversy. As in the mouse, heterologous co-expression of human SLO3 and LRRC52 results in currents that display similar [Ca^2+^]_i_ dependence, P4 sensitivity and single-channel conductance to native human sperm K^+^ current (Brenker *et al.,* 2014), suggesting that SLO3 is the mediator of human I_KSper_ (Brenker *et al.,* 2014; Mansell *et al.,* 2014). However, based mainly on the pharmacological profile, Mannowetz et al. (2013) suggested that the Ca^2+^-activated Big Potassium (BK) channel SLO1, not SLO3, is the K^+^ channel in human sperm. Lopez-Gonzalez et al. (2014) used a voltage-sensitive dye to study the effects of pharmacological manipulation of Vm and concluded that both SLO1 and SLO3 contribute to capacitation-mediated hyperpolarization. The authors suggested that a combination of SLO1 and SLO3 channels may underlie capacitation-associated hyperpolarization or even that SLO1 and SLO3 subunits may form heterotetramers with a distinct pharmacological profile. Although proteomic evidence suggests that SLO3 rather than Slo1 is the primary K^+^ channel subunit expressed in human sperm (Wang *et al.,* 2013; Brenker *et al.,* 2014), patients with loss of SLO3 function must be studied in order definitively to answer questions on the composition of the human sperm potassium channel.

Human I_Ksper_ is Ca^2+^-sensitive (see above) and P4 can elevate [Ca^2+^]_i_ to a level sufficient to potentiate I_KSper_. Reciprocally, activation of I_KSper_ hyperpolarizes Vm that may reduce CatSper opening, suggesting a connection between activity of CatSper and regulation of membrane potential (Brenker *et al.,* 2014). As high concentrations of P4 inhibit I_KSper_, it has been proposed that in the outer vestments of the cumulus-oocyte-complex high P4 levels cause hyperactivation of sperm motility by a combination of I_CatSper_ activation and I_KSper_ block (Mannowetz *et al.,* 2013).

### Evidence for and consequences of impaired potassium channel function in human sperm

Patch clamp electrophysiological analysis is the only direct way to study I_KSper_. Electrophysiological screening for K^+^ conductance abnormalities in sperm from patients who were attending for treatment, or had previously failed ART, suggested that ~10% of patients had sperm that exhibited completely depolarized Vm (≥0 mV), which was caused either by negligible I_KSper_ or enhanced inward conductance (Brown *et al.,* 2016). Despite normal CatSper function (P4-induced [Ca^2+^]_i_ increase, viscous media penetration and motility kinematics were normal), samples where sperm exhibited these abnormalities were significantly more likely to achieve ‘poor’ levels of fertilization (<25% fertilization in cycles with four or more metaphase II oocytes) than those with a more negative membrane potential. Exome analysis of one patient (patient D) whose cells had a stable lesion in outward conductance, did not reveal any causative mutations. This study reveals that abnormalities of K^+^ conductance and/or Vm regulation are both common and complex, and that fertilization potential of affected cells is severely diminished (Brown *et al.,* 2016). However, the exact nature of the impairment of function remains unknown and is an essential aspect of future investigations.

## Other ion channels in human sperm

Using whole-cell patch clamp electrophysiology, recordings of several other channels in human sperm have also been reported in the literature. Under conditions of an outward proton gradient, human sperm are shown to possess a depolarization-activated proton conductance through Hv1 ion channels, which are localized to the principal piece (Lishko *et al.,* 2010). These currents (I_HSper_) are elevated in capacitated cells and are potentiated by the endocannabinoid anandamide but blocked by Zn^2+^. The robust currents seen in human sperm contrast sharply with the extremely limited ability of mouse sperm to conduct protons. In human sperm, activation of Hv1 is proposed to facilitate capacitation through H^+^ efflux, which elevates pH_i_ and activates CatSper conductance. Therefore, functionally, Hv1 activity is permissive for development of hyperactivated motility, enhanced flagellar rotation and the acrosome reaction (Lishko *et al.,* 2010; Miller *et al.,* 2018; Zhao *et al.,* 2018). Additionally, the alkalinisation of sperm intracellular pH is essential for numerous other processes (Nishigaki *et al.,* 2014). Super-resolution imaging has demonstrated that Hv1 channels are arranged asymmetrically along the length of the principal piece in bilateral longitudinal lines in human sperm (Miller *et al.,* 2018). However, the nature of the structure and function of mature Hv1 in human sperm or during spermatogenesis is unresolved as the cells express full length and N-terminal truncated (HV1SPER) proteins (Berger *et al.,* 2017) and, *in vitro*, these proteins can form homodimers or HV1/HV1SPER heterodimers (Berger *et al.,* 2017).

Activity of calcium-dependent chloride channels (CaCC) has been recorded using patch clamp electrophysiology in human sperm. This current has properties that closely resemble heterologously expressed TMEM16A CaCC. Inhibition of CaCC effectively reduces the acrosome reaction induced by solubilized recombinant zona pellucida 3 (ZP3) protein (Orta *et al.,* 2012). Although estimations of intracellular [Cl^−^] varied considerably, (41–69 mM; Garcia and Meizel, 1999), calculated values for E_Cl_ were positive with respect to Vm of capacitated cells, such that opening of these channels will cause depolarization of the cell. In contrast, Brenker et al. (2014) did not find evidence of a Ca^2+^-sensitive chloride channel.

Cystic fibrosis transmembrane regulator (CFTR) is a protein kinase A (PKA)-activated anion channel that is localized to the equatorial segment of the sperm head in human and mouse (Xu *et al.,* 2007; Li *et al.,* 2012). Electrophysiological evidence has been presented for CFTR in mouse (Figueiras-Fierro *et al.,* 2013) but not in human sperm. Pharmacological inhibition of CFTR in human sperm using the selective inhibitor inh-172 reduces hyperactivation, the ZP3-induced acrosome reaction and cell penetration of zona-free hamster eggs (Li *et al.,* 2010; Puga Molina *et al.,* 2017). These effects have been attributed to reduced bicarbonate ion entry and lower soluble adenylate cyclase activity (Puga Molina *et al.,* 2017) that manifest as reduced sperm cAMP levels and low PKA activity. Consequently, capacitation-associated events are reduced as demonstrated by the inhibition of total protein tyrosine-phosphorylation (a general marker of capacitation) and intracellular alkalinisation (Puga Molina *et al.,* 2017). Evidence from mouse experiments supports a role for CFTR in male fecundity as CFTR heterozygous male mice exhibit reduced fertility *in vivo* and *in vitro* (Xu *et al.,* 2007). Studying the effect of CFTR mutations on human sperm fertilizing capacity is complicated due to the primary cause of infertility in upwards of 95% of affected men being congenital bilateral absence of the vas deferens (CBAVD; Chen *et al.,* 2012; de Souza *et al.,* 2018). However, a high frequency of CFTR mutations have been found in non-CBAVD males that are associated with azoospermia, oligozoospermia and oligoasthenozoospermia (Kanavakis *et al.,* 1998; Stuppia *et al.,* 2005; Schulz *et al.,* 2006; Tamburino *et al.,* 2008; Li *et al.,* 2010; Tomaiuolo *et al.,* 2011). Therefore, although complete failure of spermatogenesis is not necessarily a consequence of CFTR mutations (Stuhrmann and Dörk, 2000), they may impair optimal sperm production and maturation (Chen *et al.,* 2012). However, as there are over 2000 CFTR mutations classified into at least six classes depending on the severity of CFTR protein defect (Marson *et al.,* 2016; http://www.genet.sickkids.on.ca/cftr), any consideration of the effect of CFTR mutations on spermatogenesis and sperm function must be considered in the context of the type and zygosity of mutation. For example, individuals with the 3849+10 kb C-T mutation exhibit a mild cystic fibrosis phenotype due to the production of mutant and wild-type CFTR mRNA (Chiba-Falek *et al.,* 1999) and there is a case report of a homozygous affected man being fertile (Dreyfus *et al.,* 1996). Interestingly, IVF success has been reported using surgically retrieved sperm from men with CBAVD caused by the most severe class of CFTR mutation (Patrizio *et al.,* 1993; Schlegel *et al.,* 1995), suggesting CFTR is not an absolute requirement for IVF success. Despite the poor IVF rate, ICSI is a more effective treatment option for men with CFTR mutations and CBAVD (Schlegel *et al.,* 1995). A possible explanation for the IVF success of sperm with an assumed absence of CFTR-mediated bicarbonate entry in these cases is the activity of SLC268A anion transporter. It is expressed in human sperm and is proposed to interact with CFTR to facilitate bicarbonate entry, capacitation and normal sperm motility (Chen *et al.,* 2012; Rode *et al.,* 2012; Dirami *et al.,* 2013). If SLC268A is expressed in sperm from men with cystic fibrosis, it may partially compensate for the lack of CFTR during IVF. Considering the current evidence, there are clearly many unanswered questions regarding the role of CFTR in human sperm fertilization competence and undertaking detailed systematic studies using sperm from men with known cystic fibrosis mutations would be necessary to help answer them.

Transient receptor potential (TRP) channels form a large family of Ca^2+^ conducting channels that are sensitive to a wide variety of stimuli. TRPV1, TRPV4 and TRPM8 are immunolocalized to the head and flagella of human sperm (De Blas *et al.,* 2009; De Toni *et al.,* 2016; Kumar *et al.,* 2016). Agonist activation of these channels results in an increase in [Ca^2+^]_i_. Menthol (which opens TRPM8) induces the acrosome reaction in human sperm, although does not affect motility (De Blas *et al.,* 2009). However, it has been noted that mibefradil and NNC55-0396 block the menthol-induced increase in [Ca^2+^]_i_, suggesting menthol may activate CatSper (Brenker *et al.,* 2012). TRPV1 is proposed to mediate human sperm thermotaxis (De Toni *et al.,* 2016). TRP channels may also contribute to capacitative calcium entry (CCE, Blackmore, 1993). CCE involves the influx of extracellular calcium through plasma membrane ion channels that is triggered following the depletion of intracellular calcium stores. Stromal interaction molecule (STIM) and calcium release-activated calcium channel protein (ORAI) are key mediators of CCE in somatic cells and are also present in human sperm. Upon depletion of Ca^2+^ from intracellular stores, STIM1 translocates to the plasma membrane and activates ORAI causing a Ca^2+^ influx which is then used to refill the stores through the action of the sarcoendoplasmic reticulum calcium ion transport ATPase (SERCA) (see Correia *et al.,* 2015 for a detailed discussion of the regulation of sperm intracellular Ca^2+^ stores). Several studies provide evidence for CCE in human sperm (Lefievre *et al.,* 2012), which may explain why hyperactivation induced by 4-aminopyridine (4AP) to mobilize calcium release from intracellular stores was normal in CatSper-null sperm (Williams *et al.,* 2015). Interestingly, TRPV4 is proposed to carry a depolarizing Na^+^-conductance ‘DSper’ that facilitates calcium entry via CatSper/HV1 activation (Mundt *et al.,* 2018; Fig. 1B). However, of note is that in the absence of CatSper there is no residual conductance (Smith *et al.,* 2013; Williams *et al.,* 2015; Luo *et al.,* 2019). The explanation for this is unclear but if membrane expression of TRPV4 is CatSper-dependent, it may form part of a CatSper-dependent protein complex domain.

Finally, voltage-gated sodium channels (Na_v_ 1.2, 1.4-1.9) are also proposed to have a functional role in human sperm. They are immunolocalized to different regions of human sperm, and pharmacological activation has a positive effect on motility (Pinto *et al.,* 2009; Cejudo-Roman *et al.,* 2013). However, there is currently no supportive electrophysiological evidence for their expression.

To date, reports of definitive abnormalities of patient sperm ion channels are limited to those affecting I_CatSper_ and I_KSper_; therefore, many questions remain regarding the role(s) of these other channels in sperm function and fertilization and the incidence and consequences of their malfunction.

## Molecular and functional deficits of human sperm with channel impairments

Ion channel expression requires the successful co-ordination of a complex series of events involving transcriptional regulation, post-translational quality control, assembly, trafficking and insertion into the plasma membrane. Sequestration is governed by internalization, recycling and degradation processes. Therefore, disruption of the regulatory molecules involved in these events can culminate in defective channel expression (see Curran and Mohler, 2015 and Onwuli and Beltran-Alvarez, 2016 for examples affecting other ion channel families).

### Transcription and translation

The available evidence indicates that the dynamics of channel expression during spermatogenesis are highly complex and observations are overwhelmingly restricted to examples from mouse experiments. Transcriptional control regions and transcription factors that influence *CATSPER1* promoter activity *in vitro* have been described (Mata-Rocha *et al.,* 2013, 2014; Olivares *et al.,* 2018; Oviedo *et al.,* 2018). CatSper subunit gene expression in mice is developmentally regulated. *Catsper2*, *delta* and *epsilon* messenger RNAs are readily detected first at postnatal day 17–18 (Li *et al.,* 2007; Chung *et al.,* 2011, 2017) with significant levels of expression of *Catsper zeta*, *3*, *4* and *1* delayed until postnatal day 20–25 (Jin *et al.,* 2005; Li *et al.,* 2007; Chung *et al.,* 2017). *Efcab9* transcript expression is notably delayed with significant levels appearing between 23 and 49 days post-partum (Hwang *et al.,* 2019). Parallels in the timing of expression of mouse and human *Catsper 1* and *3* transcripts around the mid-spermatid stage have been observed (Hermann *et al.,* 2018). The reasons for, and regulation of, these temporal differences are unknown but failure to activate transcription of one subunit can prevent channel expression. For example, *Catsper delta*^−/−^ mice are infertile due to the loss of channel expression in sperm with significantly reduced CATSPER 1 protein levels reported in testis suggesting that the early expression of the DELTA subunit may help to stabilize the channel complex. In contrast, CATSPER EPSILON, but not CATSPER 3, 4, DELTA, BETA and GAMMA, protein is significantly reduced in *Catsper1*^−/−^ testis (Chung *et al.,* 2011, 2017). Indeed, CATSPER BETA and GAMMA form stable complexes in the testis of this mutant mouse strain although this does not prevent the total loss of all protein subunits from mature sperm. As discussed, functional CatSper expression in mouse sperm is contingent upon expression of all four pore-forming alpha-subunits. However, sperm from *Catsper zeta*^−/−^ and *Efcab9*^−/−^ mice express all other CatSper subunits, but at significantly reduced levels, which is reflected by reduced I_CatSper_ in these cells. Interestingly, mRNA and protein levels are unaffected in the testes of *Catsper zeta*^−/−^ mice suggesting that CATSPER ZETA prevents post-translational errors in channel assembly, the nature of which remain elusive. Intriguingly, CATSPER ZETA expression in mature sperm exhibits an interdependence with EFCAB9 expression (Chung *et al.,* 2017; Hwang *et al.,* 2019). It will be important to determine if a similar error contributes to sub-optimal P4-induced [Ca^2+^]_i_ responses in sperm from infertile patients (Alasmari *et al.,* 2013a; Kelly *et al.,* 2018). In the case of patient 1 described in Williams et al. (2015), we have suggested that loss of functional expression of CatSper may occur due to the mutant transcript being subjected to nonsense-mediated decay. Alternatively, if translation proceeds, a highly conserved methionine and alanine will be absent in the putative extracellular domain of CATSPER EPSILON. This is predicted to be highly deleterious to its conformation and to channel assembly (Brown *et al.,* 2018; Fig. 2). To understand human CatSper assembly and its failure in some men, we must continue to endeavor to understand the expression and assembly of all subunits during spermatogenesis.

**Figure 3:**
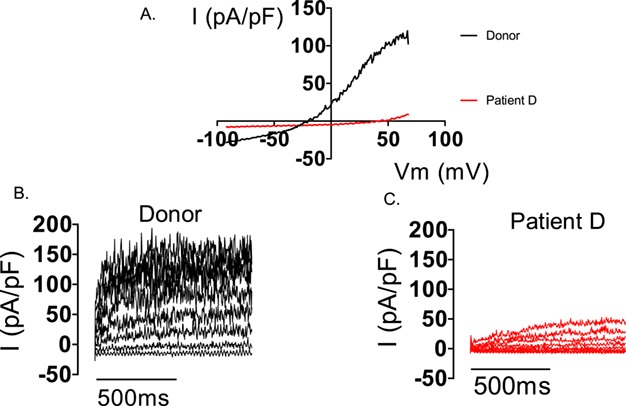
**Electrophysiological recordings from sperm from a donor and patient D (lacking I**_**KSper**_ **).** Consecutive electrophysiological recordings of a spermatozoon from a donor and patient D to ramp (−92 to 68mV, **A**) and stepped (−52 to 68 mV, **B** and **C**) depolarization under quasi-physiological ion conditions (see Brown et al., 2016). Both depolarization protocols induce a robust outward current in the donor cell. In contrast, only under extreme depolarization (>98 mV) is outward current seen in the cell from patient D (C).

In mice, messenger RNAs for SLO3 subunits and for the auxiliary subunit LRRC52 appear from around postnatal day 25. Slo3^−/−^ mice have normal mRNA expression for LRRC52 but levels of the protein in testis are significantly reduced (compared to wild-type) and undetectable in sperm. In contrast, LRRC52^−/−^ animals express SLO3 in sperm (Zheng *et al.,* 2015). These data indicate that SLO3 is necessary for the stabilization and expression of its auxiliary subunit. In cases where sperm lack I_KSper_, it will be important to determine if both channel subunits are absent or if channels composed of SLO3 only are present which exhibit severely reduced voltage sensitivity in the absence of LRRC52. For example, in the study of K^+^ channel function in infertile men (Brown *et al.,* 2016), sperm from patient D had negligible K^+^ conductance at physiological voltages, but some outward current was detectable when very high voltages (>100 mV) were imposed on the cells (Brown, unpublished observation; Fig. 3). However, at these voltages the observed outward currents might have been carried by CatSper (Brenker *et al.,* 2014).

In addition to I_CatSper_ and I_KSper_ that have been well characterized directly by whole-cell patch clamp, data from immunolocalization and pharmacological studies suggest that a number of other Ca^2+^ and K^+^ channels are expressed in male germ cells. These channels may be regulated in such a way that their activity cannot be detected in patch clamp studies (Publicover and Barratt, 2012), or they may be active only during spermatogenesis, such that antibodies can detect epitopes in mature sperm despite the fact that the channel is no longer functional. For instance, voltage-activated Ca^2+^ channel currents have been recorded in mouse *Catsper1*^−/−^ spermatocytes and mature sperm show strong positive immunoreactivity for these channels (Arnoult *et al.,* 1996; Goodwin *et al.,* 1997; Serrano *et al.,* 1999; Westenbroek and Babcock, 1999; Wennemuth, *et al.,* 2000; Ren *et al.,* 2001; Carlson *et al.,* 2003). Similarly, K_ATP_ and inwardly rectifying K^+^ currents have been recorded in mouse spermatocytes, with positive immunoreactivity for both channels reported in mature sperm (Muñoz-Garay *et al.,* 2001; Acevedo *et al.,* 2006). Intriguingly, Gong et al. (2002) suggested that temporal changes occur in the K^+^ channel population of rat spermatocytes. We previously reported that sperm from one patient, who achieved low fertilization rate at IVF, were highly depolarized and exhibited an unusually large inward (probably Na^+^) current (Brown *et al.,* 2016). Possible sources of this aberrant depolarizing current include the persistence of a sodium channel expressed in immature germ cells that is normally down regulated in the mature sperm, abnormal Na^+^ conductance through CatSper and/or K^+^ channel (both of which are permeable to Na^+^), or even activity of TRPV4, newly identified as the depolarizing current DSper (Mundt *et al.,* 2018; see above). Therefore, to enlighten understanding of the nature of channel abnormalities, future work should involve investigation into channel localization and assembly using detailed comparative proteomics combined with high quality co-localization, imaging and electrophysiology.

### Plasma membrane organization (trafficking/insertion and processing)

Cases of abnormalities in sperm ion channels that are transient or independent of deleterious channel exon mutations may reflect defects in the complex regulation of channel expression that must occur during spermatogenesis, epididymal storage and maturation or capacitation in the mature sperm. The potential for such abnormalities was recently demonstrated in a study by Chung *et al.* (2014) that used high-resolution imaging to give a novel insight into the dynamic changes that occur in the organization of CatSper in the sperm flagellum. The 3D-stochastic optical reconstruction microscopy (STORM) imaging of mouse sperm revealed that CatSper are present in a highly organized continuous quadrilateral spatial arrangement down the length of the principal piece. By imaging sperm from wild-type and *Catsper 1*^−/−^ mice using STORM, it was elegantly demonstrated that four linear ‘racing stripe’ CatSper domains co-localize with regulatory proteins of calcium signalling and spatiotemporally restrict the tyrosine phosphorylation (P-Tyr) of flagellar proteins that is necessary for development of hyperactivated motility. The P-Tyr levels in mutant sperm were unregulated, resulting in elevated phosphorylation of a wide range of proteins, including 11 structural proteins (five of which were axonemal), potentially providing molecular insight into the impaired motility of CatSper null sperm. In wild-type cells the organization of the CatSper domain was intact in cells exhibiting hyperactivated motility. However, a remarkably heterogeneous pattern of channel subunit and regulatory molecule delocalization was observed in non-hyperactivated cells during capacitation, to the extent that these domains became degraded in some cells. Therefore, the role and regulation of endocytosis of ion channels and the sperm proteasome on channel expression merit close examination. In both *Catsper zeta* and *Efcab9*-null mouse sperm, which exhibit lower channel protein expression and consequently lower I_CatSper_, the overall quadrilateral banding is maintained but the linear domains become disrupted (Chung *et al.,* 2017; Hwang *et al.,* 2019). Improved resolution of imaging of the CatSper domains suggests that each domain consists of two parallel rows of continuously expressed CatSper and loss of EFCAB9 expression results in four single row domains consisting of CatSper expressed discontinuously (Hwang *et al.,* 2019). EFCAB9 and CATSPER ZETA are notable not only because of their interdependence of expression but also their postulated functional interaction and importance for appropriate channel assembly and function. Although the stoichiometry remains unknown, CATSPER ZETA-EFCAB9 is proposed to form a pH and intracellular Ca^2+^-sensitive complex, which regulates CatSper conductance in mouse sperm. At low (pH 6) intracellular pH, the complex limits channel activity. However, alkalinisation weakens their interaction to permit calcium ion influx that can bind to Ca^2+^-binding domains of EFCAB9. This further weakens its interaction with CATSPER ZETA to maximize CatSper conductance (Hwang *et al.,* 2019). If this mechanism is paralleled in human sperm, the interplay between channel subunits and plasma membrane is likely to be more complex given that P4 or PGE1 is required to potentiate CatSper conductance. It will be important to determine if K^+^ channels are part of a regulatory complex involving CatSper, particularly given the probable interplay between these channels and evidence that loss of CatSper in human sperm may have a negative and positive impact on I_KSper_ and I_HSper_ density, respectively (Smith *et al.,* 2013). How such a specific spatial organization of CatSper is achieved is an important question that must be addressed, particularly because human CatSper is organized in the same distinct pattern (Chung *et al.,* 2017). A strategy combining imaging, proteomics and electrophysiological analysis of sperm from patients undergoing IVF will begin to address the importance of this for human fertilization.

A tantalizing insight into the processing of CatSper by the male tract was achieved by comparing I_CatSper_ in ejaculated sperm with surgically retrieved sperm from the epididymis and testis. Basal and P4-induced currents were comparable but CatSper desensitization by repeated challenge with a saturating concentration of P4 occurred only in ejaculated sperm (Smith *et al.,* 2013). The reason for this is unknown but, given the apparent complexity of the mechanism of the P4-mediated calcium influx, indirect regulation of CatSper (Mannowetz *et al.,* 2017) cannot be excluded. Indeed, beyond the characterization of P4 and PGE responses, the regulation of CatSper is largely unknown. For example, PKA is proposed to regulate mouse CatSper but it is not clear if this is due to phosphorylation of the channel (Orta *et al.,* 2018). Cysteine-rich secretory proteins (CRISP) 1 and 2 are necessary for optimal fertilization in mouse through divergent mechanisms that may involve CatSper. CRISP1 regulates sperm orientation and hyperactivation possibly through inhibitory actions on TRPM8 and CatSper (Ernesto *et al.,* 2015). IVF success is lower with cumulus-oocyte-complexes from *Crisp1*-null mice due to the loss of expression of CRISP1 from cumulus cells (Ernesto *et al.,* 2015) suggesting an extracellular-mediated mode of CatSper regulation. In contrast, heterologous mouse CRISP2 interacts with CATSPER1 *in vitro* (Lim *et al.,* 2019). *Crisp2*-deficient male mice are subfertile with sperm that have an impaired flagellar beat due to a stiff mid-piece. However, it is not established if calcium signalling is altered in mutant sperm and, if so, whether it is caused by an effect on CatSper function and/or regulation of intracellular stores (Lim *et al.,* 2019). However, given the association of reduced CRISP2 expression and asthenozoospermia (Zhou *et al.,* 2015, 2017) its potential role in human [Ca^2+^]_i_ regulation merits further study.

Finally, consideration should be given to the consequences of environmental disruption on the male reproductive tract. For example, two of the patients identified in Brown et al. (2018), who exhibited loss of I_KSper_ (patients D and K) had undergone a vasovasostomy. While this observation could be coincidental, particularly given the pregnancy rate achieved following vasectomy reversal (Majzoub *et al.,* 2017), the process of channel biogenesis is currently poorly understood and thus the implications of local trauma are genuinely unknown. Vasectomy is documented to impact on the epididymal environment, such as changes in gene expression (and potentially sperm fertilizing capacity) (Sullivan and Mieusset, 2016), and thus the effects on channel development during passage of the male tract require further investigation.

### The nature of human sperm dysfunction in sperm with ion channel defects (molecular and physiological)

The effects of ion channel dysfunction on human sperm function are likely to be diverse both in their nature and severity. Clearly, if a lack of CatSper function results in severely impaired adaptive motility, this is likely to have a profound and complete impairment on fertility. The fact that this phenotype is extremely rare is consistent with this conclusion. However, the effects of changes in voltage sensitivity, ion selectivity or agonist sensitivity are less easy to predict. Similarly, the effects of K^+^ conductance may vary widely (Brown *et al.,* 2016) depending on the size and nature of the remaining I_KSper_ and how this affects activity of CatSper and (probably) other ion channels. Similarly, effects on downstream protein phosphorylation could potentially affect a multitude of interconnected signalling pathways that determine sperm fertilizing capability. Defects may manifest as impairment(s) of motility, reduced or absent binding/interaction with the female tract and the cumulus-oocyte complex or failed acrosome reaction (Luo *et al.,* 2019). These effects, alone or in combination, could explain the failed fertilization at IVF that is characterized by defective [Ca^2+^]_i_ responses to P4 in human sperm (Krausz *et al.,* 1995; Williams *et al.,* 2015; Luo et al. 2019).

To fully understand the effects of sperm channel dysfunction on human fertility and develop effective treatments for male infertility, we need to study the activity and function of sperm in the context of the human female tract. However, this is currently impossible. A priority must therefore be the development of experimental models that reconstitute, as closely as possible, the *in vivo* environment. For example, remarkable work with microfluidic 3D cultures of murine ovarian and human tissues has led to the culture of an *ex vivo* female reproductive tract model that can mimic a 28-day human menstrual cycle (Xiao *et al.,* 2017). The female tract, through which the sperm must navigate to reach the egg, presents a range of topographical and physiochemical challenges. *In vitro* experiments demonstrate that sperm motility is adaptive and complex. For example, human sperm preferentially swim along the wall of microchannels (Denissenko *et al.,* 2012) and are observed to exhibit an intermittent 2D motility pattern that is influenced by viscosity (Nosrati *et al.,* 2015) and fluid flow (Zhang *et al.,* 2016). Furthermore, 3D movement patterns are multiform (Su *et al.,* 2012, 2013). Sperm exposed to fluid flow *in vitro* will swim upstream (rheotaxis), a CatSper-dependent response in mouse sperm that is believed to direct swimming up the oviduct, against the flow of oviductal fluid (Miki and Clapham, 2013; Zhang *et al.,* 2016; Chung *et al.,* 2017). The importance of this *in vivo* has recently been questioned given the observed isthmus-to-ampulla peristaltic movement of the mouse oviduct and rapid movement of oviductal fluid. It was argued that these, along with the cell’s motility, are sufficient for progressing sperm towards the ampulla (Hino and Yanagimachi, 2019). Nevertheless, human sperm are capable of rheotaxis (Kantsler *et al.,* 2014; Bukatin *et al.,* 2015) although in contrast to mouse sperm, it has been proposed to be passive (Zhang *et al.,* 2016) arguing against CatSper involvement. It is possible that sperm participate actively in their navigation, using both thermotaxis (Boryshpolets *et al.,* 2015; De Toni *et al.,* 2018) and chemotaxis (Armon *et al.,* 2014; Perez-Cerezales *et al.,* 2015) to modify their motility and behavior in response to cues from the physically and chemically complex environment of the female tract and cumulus-oocyte complex (Borland *et al.,* 1980; Zhao and Chegini, 1994; Adachi *et al.,* 1995; Tay *et al.,* 1997; Schuel *et al.,* 2002; Aguilar and Reyley, 2005). Moreover, the development of hyperactive motility may assist in the release of sperm after they have bound to the epithelial lining of the tract (Pacey *et al.,* 1995). *In vitro* studies suggest that ion channels are central to the control of sperm motility and behaviour (Correia *et al.,* 2015).

It is likely that the context in which effects of ion channel function are assessed is critical. For example, in experiments with human follicular fluid (hFF) the concentration of P4 and consequent effects on I_CatSper_ was the primary determinant of the observed effects [Ca^2+^]_i_ responses and motility kinematics. However, it appeared that other components of hFF modulated [Ca^2+^]_i_ signalling (Brown *et al.,* 2017). Another interesting observation highlighting the necessity of physiological context was reported following experiments in *Catsper zeta*-null mice. *Catsper zeta*-null male mice show only low levels of fertilization and epididymal CATSPER ZETA-null sperm fail to fertilize *in vitro*. However, when ejaculated sperm retrieved from the uterus were used for IVF, fertilization was partially rescued (Chung *et al.,* 2017). The mechanism of this phenomenon is unknown, nor is it clear if it occurs due to components added in the ejaculate (as suggested to occur in humans, Park *et al.,* 2011) or present in the female tract (see also Li *et al.,* 2015).

Perhaps, the most surprising finding from recent studies of sperm samples from patients that exhibited ion channel dysfunction was that basal motility in cells incubated in capacitating conditions (including the proportion of hyperactivated cells) in both I_CatSper_ and I_KSper_ null sperm was within the normal range (assessed by CASA). However, whereas I_KSper_ null sperm were stimulated by P4 to penetrate viscous medium (≥100% increase compared to control), stimulation of I_CatSper_ null sperm with P4 had no effect (Williams *et al.,* 2015). The implication of this is that either ion channel dysfunction does not impact on basal flagellar motility patterns or that current CASA systems may not be sufficiently discriminatory to detect important differences in flagellar beat and behavior of cells with ion channel lesions. Future experiments must employ advanced methods of flagellar or behavioral analysis and cell tracking (Su *et al.,* 2012, 2013; Brown *et al.,* 2016).

## Final perspective

Patch clamp electrophysiology has revolutionized our understanding of ion channel function in both the healthy and dysfunctional spermatozoon. Indeed, from our studies of sperm samples from patients, we hypothesize that loss of I_CatSper_ and I_KSper_ can cause male subfertility. However, identification of causal defects (genetic and non-genetic) and the precise molecular and functional impairments that arise as a consequence, remains a significant but exciting challenge. Development of new technologies is critical, both to illuminate our understanding as well as facilitating progress towards the development of routine diagnostic tests and individualized medicine to advance patient treatment.

Reproductive physiology is shaped by selective processes. During natural fertilization, the male and female tracts assist in preparing (Park *et al.,* 2011; Reilly *et al.,* 2016) and selecting the most viable and, presumably, genetically fit spermatozoa to produce healthy offspring. In contrast, ART uses invasive, *in vitro* techniques to enable malfunctioning gametes to fertilize the oocyte. A primary aim of research in reproductive medicine should be, where possible, to restore normal gamete function so that these selection mechanisms can operate and fertilization can occur naturally (or as near naturally as possible). This is a major challenge, not least because of our dearth of current understanding (see, for example, De Jonge, 2017). Arguably, significant acceleration and advancement of male reproductive medical research necessitates a concerted international collaborative effort involving all relevant stake holders (D’Hooghe, 2017; Barratt *et al.,* 2018; Brown *et al.,* 2018). This is a huge challenge and perhaps more difficult owing to the fact that our discipline is unique in that diametrically opposed success outcomes are sought for sperm by those seeking assisted conception and those requiring contraception (see Barratt *et al.,* 2018 for an argument for the case for development of a male contraceptive).

However, comprehension of human spermatogenesis, sperm behaviours and fertilization at a molecular level will advance both causes. Here we have argued that understanding the expression and functioning of human sperm ion channels is necessary for the development of improved therapies for male infertility but equally recent studies show that compounds that inhibit I_CatSper_ and I_KSper_ may make effective contraceptives. The sperm-specific nature of I_CatSper_ and I_KSper_ (see Lishko, 2016 for additional examples) makes them particularly suitable targets for compounds that can even be taken by the female, thus circumventing concerns of non-compliance of the man (Glasier *et al.,* 2000).

It is clearly critical to observe and understand the mysteries of sperm *in vivo*. Recent investigations have used transgenic mouse sperm that express fluorescent markers to permit live imaging *in/ex vivo* (Hino *et al.,* 2016; Muro *et al.,* 2016). These studies gave remarkable novel insights into sperm behaviour and we echo the sentiment expressed by Muro et al. (2016) by concluding that what is yet to be discovered about *in vivo* fertilization in humans is currently beyond our imagination but argue that sperm plasma membrane ion channels will play a central role.
